# GDF10 is related to obesity as an adipokine derived from subcutaneous adipose tissue

**DOI:** 10.3389/fendo.2023.1159515

**Published:** 2023-07-14

**Authors:** Mi Kyung Song, Ji Eun Kim, Jung Tae Kim, Yea Eun Kang, Sun Jong Han, Seok Hwan Kim, Hyun Jin Kim, Bon Jeong Ku, Ju Hee Lee

**Affiliations:** ^1^ Department of Internal Medicine, Chungnam National University College of Medicine, Daejeon, Republic of Korea; ^2^ Research Center for Endocrine and Metabolic Disease, Chungnam National University College of Medicine, Daejeon, Republic of Korea; ^3^ Department of General Surgery, Chungnam National University College of Medicine, Daejeon, Republic of Korea

**Keywords:** GDF10, subcutaneous fat, adipokines, obesity, adipose tissue

## Abstract

**Introduction:**

Adipokines are proteins that are secreted by the adipose tissue. Although they are associated with obesity-related metabolic disorders, most studies have focused on adipokines expressed by visceral adipose tissue (VAT). This study aimed to identify the adipokine potentially derived from subcutaneous adipose tissue (SAT) and its clinical significance.

**Methods:**

Samples of SAT and VAT were obtained from six adult male patients who underwent laparoscopic surgery for benign gall bladder disease. Differentially expressed genes were analyzed by subjecting the samples to RNA sequencing. The serum concentration of selected proteins according to body mass index (BMI) was analyzed in 58 individuals.

**Results:**

*GDF10* showed significantly higher expression in the SAT, as per RNA sequencing (fold change = 5.8, adjusted *P* value = 0.009). Genes related to insulin response, glucose homeostasis, lipid homeostasis, and fatty acid metabolism were suppressed when *GDF10* expression was high in SAT, as per genotype-tissue expression data. The serum GDF10 concentration was higher in participants with BMI ≥ 25 kg/m^2^ (n = 35, 2674 ± 441 pg/mL) than in those with BMI < 25 kg/m^2^ (n = 23, 2339 ± 639 pg/mL; *P* = 0.022). There was a positive correlation between BMI and serum GDF10 concentration (r = 0.308, *P* = 0.019).

**Conclusions:**

*GDF10* expression was higher in SAT than in VAT. Serum GDF10 concentration was high in patients with obesity. Therefore, GDF10 could be a SAT-derived protein related to obesity.

## 1 Introduction

Obesity is a chronic disease caused by excessive or abnormal accumulation of adipose tissue, resulting from a multifactorial etiology that includes genetic, environmental, and psychosocial factors. The increasing prevalence rates of overweight and obesity have become a pressing public health challenge that results in a severe global health burden (1). Despite various efforts to reduce obesity, currently available drugs focus on reducing energy intake through the inhibition of fat absorption and appetite reduction. However, obesity has many heterogeneous pathophysiological aspects, including insulin resistance and metabolic syndrome. Accordingly, it is necessary to find ways to resolve insulin resistance by understanding the surrounding environment, especially white adipose tissue.

White adipose tissue is anatomically divided into visceral adipose tissue (VAT), which is located within the abdominal cavity, and subcutaneous adipose tissue (SAT). Both tissues exhibit functionally and metabolically distinct characteristics (2). SAT shows higher insulin sensitivity and lower rates of lipolysis and free fatty acid release compared to VAT (3). The accumulation of VAT is associated with a higher risk of obesity-related complications, such as cardiovascular disease, atherosclerosis, hyperlipidemia, hypertension, and type 2 diabetes mellitus (4, 5). The differences between SAT and VAT are contributed by variations in gene expression (6, 7).

Adipose tissues are also endocrine organs that secrete a number of peptide hormones, such as leptin and adiponectin. Adipokines are related to obesity-related metabolic disorders including fat metabolism, insulin sensitivity, and energy homeostasis (8, 9). Besides the structural and functional differences between VAT and SAT, these tissues also show differential expression of adipokines (10–12). However, previous studies have mainly focused on adipokines expressed in VAT (13–15). In this study, we aimed to investigate the differential genetic profiles of SAT and VAT and identify the adipokine derived from SAT. Furthermore, we aimed to evaluate the correlation of serum concentration of the novel depot-specific adipokine with the clinical phenotype. Overall, our analysis provides a basis for developing new therapeutic targets for obesity.

## 2 Methods

### 2.1 Participants for adipose tissue biopsy

Patients who were scheduled for elective surgery at the Department of Surgery of Chungnam National University Hospital, Daejeon, Republic of Korea were prospectively enrolled. The Institutional Review Board of this hospital approved the study (approval number: 2020-02-004), and written informed consent was obtained from all participants. The study was performed in accordance with the principles of the Declaration of Helsinki. The inclusion criteria were age >19 years and elective surgery for gallbladder stone and gallbladder polyp. Individuals with untreated cancer, inflammation of the abdominal cavity, severe cardiovascular disease, cerebrovascular disease, sepsis, and uncontrolled acute inflammation were excluded. We only enrolled males for tissue biopsy to eliminate the effect of estrogen due to the menstrual cycle or menopausal status. Patients on anti-obesity medication within the past 3 months or any other clinical trial drug within the past 1 month and those with a history of drug or alcohol abuse within the past 1 year were excluded. Finally, six patients were included in this study.

The blood samples for assessing serum glucose and insulin concentration were collected from the participants when they were fasting. VAT samples were collected from the distal portion of the greater omentum during surgery under general anesthesia. SAT samples were collected from the port insertion site for laparoscopic surgery under general anesthesia. Adipose tissue specimens were snap-frozen in liquid nitrogen and stored at −70°C.

### 2.2 RNA isolation and quantitative real-time polymerase chain reaction

The tissues were homogenized using a TissueLyser II (Qiagen, Hilden, Germany), and RNA was isolated using TRIzol™ Reagent (15596018, Life Technologies, Thermo Fisher Scientific, Waltham, MA, USA). Complementary DNA (cDNA) was synthesized from the RNA (1 μg) using Oligo(dT)_15_ primer (C1101, Promega, Madison, WI, USA) and M-MLV reverse transcriptase (28025, Thermo Fisher Scientific). The produced cDNA was then amplified on a 7500 Fast Real-Time PCR System (Applied Biosystems, Carlsbad, CA, USA). Real-time PCR was performed using SFC Green Fast qPCR Master w/w ROX (2×; #1303, Taomics, South Korea). Primer specificity was verified with NCBI Primer-BLAST (https://www.ncbi.nlm.nih.gov/tools/primer-blast/), and a single peak with proper amplification efficiency in each melting curve was identified. Relative quantification was performed using the ΔΔCT method with the Applied Biosystems 7500 Software (ver. 2.0.6). Gene expression was normalized to that of 18s rRNA. The primer sequences were as follows: *GDF10*: 5′-AGATCGTTCGTCCATCCAACC-3′ and 5′-GGGAGTTCATCTTATCGGGAACA-3′; *SERPINA5*: 5′-ATGCCCTTTTCACCGACCTG-3′ and 5′-TGCAGAGTCCCTAAAGTTGGTAG-3′; *18s*: 5′-CTGGTTGATCCTGCCAGTAG-3′ and 5′-CGACCAAAGGAACCATAACT-3′.

### 2.3 RNA sequencing

Total RNA concentration was calculated by Quant-IT RiboGreen (#R11490, Invitrogen, Waltham, MA, USA). To assess the integrity of the total RNA, samples were run on the *TapeStation RNA screentape (#5067-5576, Agilent Technologies, Santa Clara, CA, USA).* Only high-quality RNA preparations with RIN greater than 7.0 were used for RNA library construction.

A library was independently prepared with 1 μg of total RNA for each sample using Illumina TruSeq Stranded mRNA Sample Prep Kit (#RS-122-2101, Illumina, Inc., San Diego, CA, USA). The first step in the workflow involves the purification of the poly-A-containing mRNA molecules using poly‐T‐attached magnetic beads. After purification, the mRNA was fragmented into small pieces using divalent cations under elevated temperatures. The cleaved RNA fragments were copied into first-strand cDNA using SuperScript II reverse transcriptase (#18064014, Invitrogen) and random primers. This step was followed by second-strand cDNA synthesis using DNA Polymerase I, RNase H, and dUTP. These cDNA fragments then underwent an end repair process, addition of a single “A” base, and ligation of the adapters. The products were then purified and enriched with PCR to create the final cDNA library.

The libraries were quantified using KAPA Library Quantification kits for Illumina Sequencing platforms according to the qPCR Quantification Protocol Guide (#KK4854, KAPA BIOSYSTEMS, Wilmington, MA, USA) and qualified using the TapeStation D1000 ScreenTape (#5067-5582, Agilent Technologies). Indexed libraries were then submitted to Illumina NovaSeq (Illumina, Inc.), and paired-end (2×100 bp) sequencing was performed by Macrogen Incorporated. After removing the low-quality and adapter sequences using Trimmomatic, the reads were aligned with *Homo sapiens (GRCh38)* using HISAT (ver. 2.1.0) (16). Two types of indices were downloaded from the UCSC table browser (http://genome.ucsc.edu) and used for alignment: a global, whole-genome index and tens of thousands of small local indices.

StringTie v2.1.3b was used to assemble the transcript (17, 18). It provided the relative abundance estimates as fragments per kilobase of exon per million fragments mapped (FPKM) values of the transcript or gene. After excluding the genes with one more than zero FPKM values, the signal value (FPKM+1) was transformed to a base 2 logarithm and normalized by quantile normalization methods to reduce systematic bias. These values were used for the analysis of differentially expressed genes (DEGs) in the adipose tissue groups. The statistical significance of the DEG values was calculated using independent t-tests (*P* < 0.05) and fold change (|FC|≥2). The false discovery rate, which estimates the frequency of type I statistical errors, was determined by adjusting the *P* value using the Benjamini–Hochberg algorithm.

### 2.4 Gene ontology and pathway-enrichment analysis with the genotype-tissue expression database

All the available GTEx data for SAT (n = 663) were obtained from the GTEx portal (https://gtexportal.org). The transcripts per million (TPM) data used for our analyses were obtained from the database of GTEx Analysis V8 (dbGaP Accession phs000424. 8.02). Of the 663 SAT samples in the GTEx data, those with *GDF10* expression in the highest (n = 165) and lowest (n = 165) quartiles were used for Gene Ontology (GO) and Kyoto Encyclopedia of Genes and Genomes (KEGG) pathway analysis. Differentially expressed genes were identified by the establishment of two groups based on *GDF10* expression with the R package DESeq2 (19). The gene-set collection of KEGG was obtained from Enrichr (https://amp.pharm.mssm.edu/Enrichr/), and gene-set enrichment analysis was conducted with the R package Platform for Integrative Analysis of Omics (PIANO) data. *P* values were adjusted using Benjamini–Hochberg correction for controlling the false-discovery rate, and the results were considered statistically significant when adjusted *P* values were < 0.05.

### 2.5 Participants involved in the measurement of serum GDF10

Patients from the outpatient clinic of the Division of Endocrinology and Metabolism of Chungnam National University Hospital (Daejeon, Korea) between March 2014 and December 2019 were enrolled in this study. The inclusion criteria were as follows: age 20–60 years, no history of medication with antidiabetic drug or drug abuse, no pregnancy, and absence of any clinical signs of infection or inflammation. Individuals with untreated malignancies, liver cirrhosis, or an estimated glomerular filtration rate of < 60 mL/min were excluded. Finally, 33 participants with newly diagnosed type 2 diabetes mellitus and 25 control participants with normal glucose tolerance were included in this analysis. All participants underwent physical examinations on the day on which the study commenced. Height and body weight were determined without shoes. Body mass index (BMI) was calculated as weight in kilograms (kg) divided by height in meters squared (m^2^). The participants were divided into two groups according to BMI: obese (≥ 25 kg/m^2^) and non-obese (< 25 kg/m^2^), according to the World Health Organization Asia-Pacific Obesity Classification. Our experimental protocol was performed in accordance with the Declaration of Helsinki. The Institutional Review Board of Chungnam National University Hospital (approval number: 2014-12) approved the protocol for this research, and written informed consent was obtained from all the participants. The biospecimens and data used for this study were provided by the Biobank of Chungnam National University Hospital.

### 2.6 Biochemical measurements

All blood samples were collected in the morning after an overnight fast of > 8 h. We measured the levels of fasting glucose, C-peptide, insulin, triglycerides (TGs), total cholesterol, low-density lipoprotein cholesterol (LDL-C), high-density lipoprotein cholesterol (HDL-C), aspartate aminotransferase (AST), alanine aminotransferase (ALT), blood urea nitrogen, and creatinine. Blood chemistry and lipid profiles were measured using a blood chemistry analyzer (TBA-2000FR; Toshiba, Otawara, Japan). Insulin was quantified using a chemically induced fluorescence immunoassay (ADVIA Centaur XP; Siemens, Erlangen, Germany). The homeostasis model assessment of insulin resistance (HOMA-IR) was calculated as follows: fasting insulin level (mIU/L) × fasting glucose level (mg/dL)/405. Serum GDF10 concentration was measured using a commercially available quantitative sandwich enzyme-linked immunosorbent assay (ELISA) kit (Catalog No. MBS2507164; MyBioSource, San Diego, CA, USA).

### 2.7 Statistical analysis

Statistical analyses were performed using SPSS software version 26.0 (IBM, Armonk, NY, USA). Continuous variables were tested for normality with the Kolmogorov–Smirnov test. Clinical data were expressed as the mean ± standard deviation (SD), and the significance of between-group differences was evaluated using the student t-test or Mann–Whitney U test, depending on the normality of distribution. The mRNA levels are expressed as mean ± standard error of the mean. The strengths of the relationships between serum GDF10 concentration and clinical parameters were analyzed by Pearson’s or Spearman correlation, depending on the normality of distribution. *P* < 0.05 was considered to represent statistical significance.

## 3 Results

### 3.1 Differentially expressed genes from RNA sequencing

**Figure 1:**
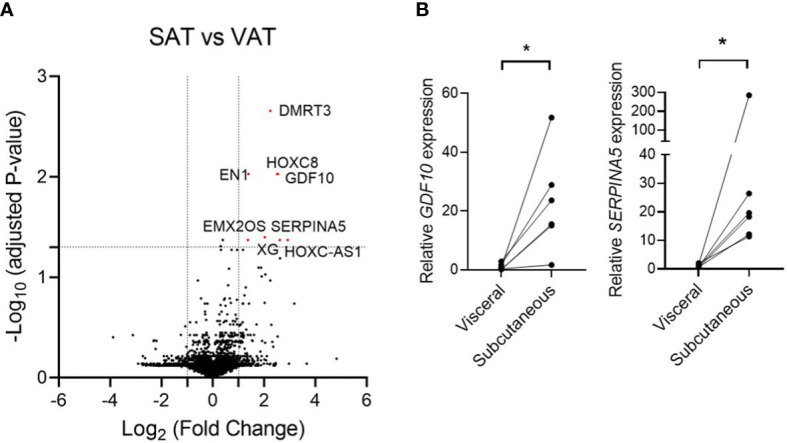
**(A)** Volcano plot of the differentially expressed genes from RNA sequencing data that compared subcutaneous adipose tissue and visceral adipose tissue. **(B)** Relative mRNA expression of *GDF10* and *SERPINA5* in subcutaneous and visceral adipose tissue. * < 0.05 by paired t-test.

The data of patients enrolled for adipose tissue biopsy for RNA sequencing are shown in **Supplementary Table 1**. The average age, BMI, and HOMA-IR of enrolled participants were 52.7 ± 6.2 years, 23.7 ± 1.9 kg/m^2^, and 1.67 ± 0.6, respectively. When we analyzed the data by considering *P* < 0.05 as being statistically significant, 121 genes were upregulated and 66 genes were downregulated in SAT compared to those in VAT (**Supplementary Data 2**). On the contrary, when we applied the *P* value that was adjusted using the Benjamini–Hochberg algorithm, only eight genes were significantly upregulated including the HOXC cluster antisense RNA 1 (encoded by *HOXC-AS1*), serpin family A member 5 (encoded by *SERPINA5*), growth differentiation factor 10 (encoded by *GDF10*), homeobox C8 (encoded by *HOXC8*), doublesex and mab-3 related transcription factor 3 (encoded by *DMRT3*), Xg glycoprotein (encoded by *XG*), engrailed homeobox 1 (encoded by *EN1*), and EMX2 opposite strand (encoded by *EMX2OS*) (**Figure 1A**). Among the top three genes with the highest fold change, *SERPINA5* (fold change = 6.1, adjusted *P* value = 0.042) and *GDF10* (fold change = 5.8, adjusted *P* value = 0.009) are protein-coding genes, while *HOXC-AS1* (fold change = 7.5, adjusted *P* value = 0.042) is a long non-coding RNA. We also confirmed the increased mRNA levels of *GDF10* and *SERPINA5* in the obtained tissues *via* quantitative real-time PCR (**Figure 1B**).

### 3.2 GTEx gene-set-enrichment analysis of SAT in relation to *GDF10* expression

To gain further insights into SAT-derived adipokines, we examined the expression of *GDF10* and *SERPINA5* in various tissues using data from the GTEx database (https://gtexportal.org). *GDF10* was more highly expressed in SAT (median TPM 18.18) than in VAT (median TPM 2.152), in terms of bulk tissue gene expression. However, the expression level of *SERPINA5* was not distinguishable between SAT (median TPM 4.892) and VAT (median TPM 1.300). SERPINA5 was expressed at higher levels in the adrenal gland (median TPM 491.1) and testis (median TPM 496.1) than in the adipose tissue. Therefore, we selected GDF10 as a possible adipokine derived from SAT.

**Figure 2:**
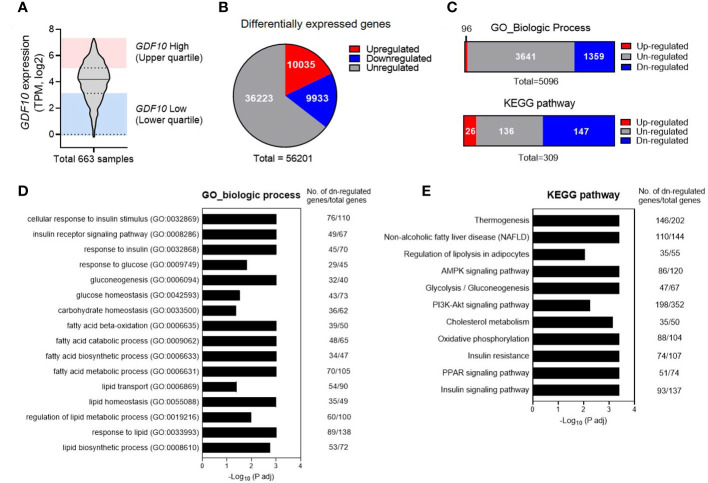
Downregulated physiological pathways in subcutaneous adipose tissue with high expression of growth differentiation factor 10 (*GDF10*) in the Genotype-Tissue Expression Database. **(A)** *GDF10* expression levels in 663 human subcutaneous adipose tissue samples in the UCSC database. **(B)** Numbers of differentially expressed genes in a comparison of the samples in the highest quartile for *GDF10* expression (n = 165) and those in the lowest quartile (n = 165). **(C)** Association of differential *GDF10* expression with Gene Ontology (GO) biological processes and Kyoto Encyclopedia of Genes and Genomes (KEGG) pathways. **(D, E)** Downregulated biological processes in the GO annotation or KEGG pathway analysis. TPM, transcripts per million.

Next, using the GTEx database, gene-enrichment analysis was performed to determine the relationship between energy metabolism and *GDF10* expression in human SAT. Among 663 SAT samples from the GTEx database, the samples with *GDF10* expression levels in the highest quartile (n = 165) and in the lowest quartile (n = 165) were used for GO and KEGG pathway analyses (**Figure 2A**). In these samples, 10,035 genes were upregulated in the high-*GDF10* group compared to those in the low-*GDF10* group (**Figure 2B**). In the GO-biological process analysis, 1359 processes were downregulated in the high-*GDF10* group compared to those in the low-*GDF10* group (**Figure 2C**). The significantly downregulated processes included the cellular response to insulin stimulus and carbohydrate, glucose, and lipid homeostasis (**Figure 2D**). In the KEGG analysis, 147 pathways were downregulated in high-*GDF10* group compared to those in the low-*GDF10* group, including pathways related to the oxidative phosphorylation, glycolysis and gluconeogenesis, and insulin signaling pathway (**Figure 2E**). GTEx database analysis showed that the genes related to insulin response, glucose homeostasis, lipid homeostasis, and fatty acid metabolism were suppressed when the *GDF10* expression was high in SAT.

### 3.3 Comparison of serum levels of GDF10 in relation to BMI

**Figure 3:**
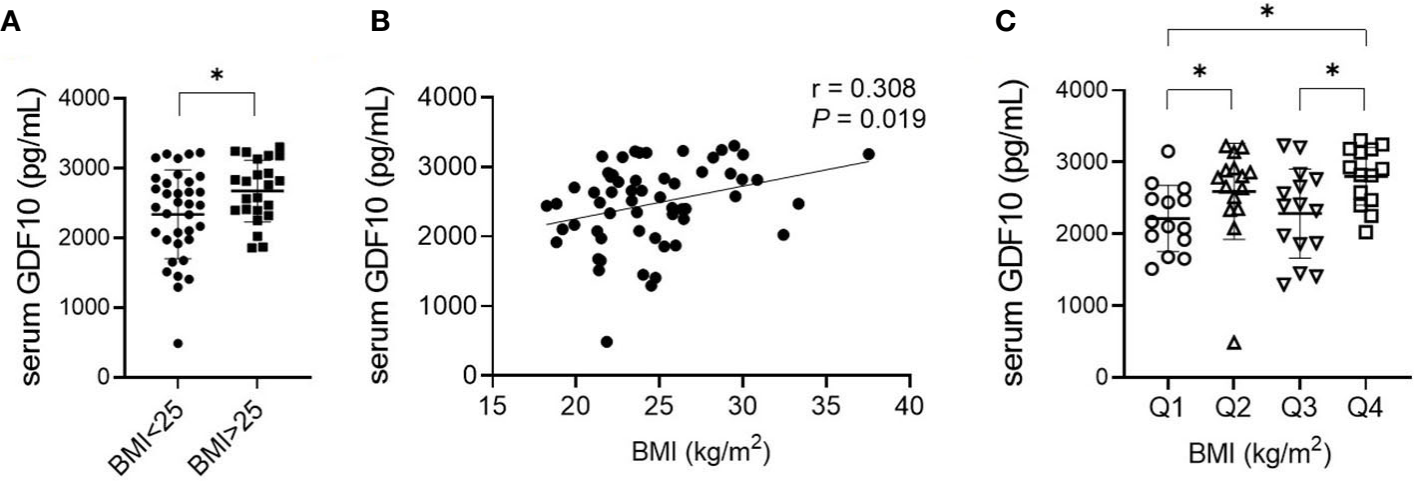
**(A)** Comparison of serum GDF10 levels in relation to BMI. *P < 0.05 by the independent Student’s T-test. **(B)** Correlation between serum GDF10 and BMI by Pearson correlation coefficient. BMI, body mass index. **(C)** Comparison of serum GDF10 levels in relation to quartile groups stratified according to BMI. *P < 0.05 by the Mann–Whitney U test.

In total, 58 individuals were enrolled and divided into non-obese (BMI < 25 kg/m^2^) and obese groups (BMI ≥ 25 kg/m^2^). The baseline characteristics of the participants are shown in **Table 1**. There were no statistically significant differences between the groups in terms of age, sex, and levels of fasting blood glucose, total cholesterol, LDL-C, AST, and eGFR. The average BMI of the non-obese group was 22.1 kg/m^2^ (range, 18.2–24.8), and the average BMI of the obese group was 28.3 kg/m^2^ (range 25.0–37.5) (*P* < 0.001). Fasting blood insulin and ALT levels and HOMA-IR were higher in the obese group, indicating a greater prevalence of insulin resistance and of non-alcoholic fatty liver disease compared to those in the non-obese group. Additionally, the obese group had a lower HDL-C level. The serum GDF10 concentration was significantly higher in the obese group than in the non-obese group (2674 ± 441 pg/mL, range 1859–3305 *vs*. 2339 ± 639 pg/mL, range 490–3225 pg/mL, respectively, *P* = 0.022) (**Figure 3A**). In the correlation analysis, serum GDF10 level was positively correlated with BMI (r = 0.308, *P* = 0.019) (**Figure 3B**) but not with age, fasting blood glucose and insulin levels, and HOMA-IR. When the subjects were stratified into quartiles based on BMI, there were significant differences in serum GDF10 levels between the first (2214 ± 460 pg/mL, range 1517–3151) and second quartiles (2593 ± 668 pg/mL, range 490–3225), between the third (2283 ± 621 pg/mL, range 1296–3231) and fourth quartiles (2803 ± 402 pg/mL, range 2026–3305), and between the first and fourth quartile (**Figure 3C**). We also compared the serum GDF10 level between normal glucose tolerance (n = 25, 2451 ± 614 pg/mL) and newly diagnosed diabetes mellitus subgroups (n = 33, 2488 ± 577 pg/mL); however, no difference was observed.

**Table 1:** Baseline characteristics of the study participants for serum GDF10 concentration.

Variables	BMI < 25 kg/m^2^ (n = 35)	BMI ≥ 25 kg/m^2^ (n = 23)	*P* value
Age, yr	45.5 ± 7.6	46.9 ± 8.9	0.479
Sex (Male : Female)	10:25	9:14	0.402
BMI, kg/m^2^	22.1 ± 1.8	28.3 ± 3.1	< 0.001
Fasting blood sugar, mg/dL	135.8 ± 66.5	153.1 ± 63.1	0.087
Fasting blood insulin, mIU/L	8.4 ± 4.8	12.9 ± 4.8	< 0.001
HOMA-IR	2.9 ± 2.0	4.9 ± 3.1	0.002
Triglycerides, mg/dL	142.7 ± 128.3	162.4 ± 97.7	0.072
Total cholesterol, mg/dL	199.5 ± 39.7	207.0 ± 41.6	0.373
LDL-C, mg/dL	120.5 ± 37.4	131.9 ± 39.2	0.248
HDL-C, mg/dL	57.3 ± 15.3	48.6 ± 10.7	0.021
AST, IU/L	22.0 ± 9.9	24.4 ± 12.2	0.331
ALT, IU/L	20.6 ± 10.8	28.6 ± 15.1	0.025
eGFR, mL/min	113.7 ± 24.1	113.2 ± 22.3	0.941
GDF10, pg/mL	2339 ± 639	2674 ± 441	0.022

Data are expressed as the mean ± standard deviation or n. P value was calculated by independent Student t-test or the Mann–Whitney U test. BMI, body mass index; HOMA-IR, homeostasis model assessment-insulin resistance; LDL-C, low density lipoprotein cholesterol; HDL, high density lipoprotein cholesterol; AST, aspartate aminotransferase; ALT, alanine aminotransferase; eGFR, estimated glomerular filtration rate; GDF10, growth differentiation factor 10.

## 4 Discussion

Adipose tissue showed characteristics that varied according to the regional depot, in terms of systemic energy metabolism and inflammatory response. The inherent properties of adipose cells as well as the extrinsic factors including hormonal and paracrine microenvironment, local nutrient availability, innervation, and anatomic constraints contribute to the depot-specific character of fat (6, 7). Several studies have attempted to validate the differences in the genetic expression of adipokines based on adipose tissue depots in humans using microarray (20–23). Vohl et al. analyzed the gene expression profiles of adipose tissue in the abdominal subcutaneous wall and omental adipose tissue in 10 obese men with a mean BMI of 59.1 kg/m^2^ using microarray (20). In another study, the omental adipose tissue and SAT from the abdominal subcutaneous wall samples collected from five overweight women with a mean BMI of 28.97 kg/m^2^ (21) were subjected to microarrays; *GDF10* level was found to be significantly decreased in VAT compared to that in SAT, as confirmed by real-time PCR, consistent with the findings of our study. On the other hand, Ahn et al. revealed 414 highly DEGs (log2 FC ≥3) including 262 protein-coding genes between subcutaneous and omental adipose depots using the database of GTEx Analysis V7. Among them, *HOXC-AS* and *DMRT3*, but not *GDF10*, showed differentially increased expression in SAT (24). The different results among the studies may be due to differences in analytical tools, samples obtained independently or as pairs, and characteristics of the study participants such as race, sex, and BMI.

Here, we performed RNA sequencing, which is a more advanced method than microarray to analyze gene expression (25). In addition, we sorted the DEGs, applied both |FC|≥2 and adjusted *P*-value, and presented the result as a volcano plot. Moreover, the paired adipose tissue was collected from Asian patients with relatively low BMI (mean 23.7 kg/m^2^), while the three studies mentioned above were performed in Western countries. As a result, we found that the expression of *GDF10* was highly increased in SAT than in VAT.

GDF10 is a TGF-β family member related to bone morphogenetic protein-3 (BMP3) (26); therefore, it is also known as BMP3B. However, *GDF10* is expressed in mammalian adipose tissue as well as in the bone and developing embryo (27, 28). In a previous study, *GDF10* expression was found to be higher in the mesenteric adipose tissue of diet-induced obesity mice than in control mice (29). Furthermore, circulating GDF10 levels were increased in mice with diet-induced hepatic steatosis (30). GDF10 attenuates the activity of nuclear peroxisome proliferator-activated receptor γ (PPARγ), a transcription factor known to induce *de novo* lipogenesis in cultured hepatocytes (30). Compared with wild-type mice, mice with adipocyte-specific BMP3B overexpression showed a decrease in weight gain, fat-pad mass, and adipocyte area when maintained on a high-fat diet (31). In our study, we found increased serum GDF10 concentration in participants with obesity, consistent with the results of the two studies performed on mice. Furthermore, increased *GDF10* expression in SAT was associated with the suppression of glucose and lipid homeostasis according to the GTEx data, although the serum GDF10 concentration did not show any difference with respect to the glucose level. Therefore, GDF10 could be an adipokine related to the pathophysiology of obesity.

We further investigated the metabolic effect of GDF10 in human adipose tissue using the GTEx data. SAT samples with higher *GDF10* expression were associated with the downregulation of several biologic processes, including insulin response, glucose homeostasis, and fatty acid metabolism, compared to those with lower *GDF10* expression. Notably, increased BMP3B expression in adipocytes inhibited the differentiation of adipocytes *in vitro* (31). Numerous KEGG pathways, including AMPK signaling, PPAR signaling, and insulin signaling pathway were also downregulated in SAT samples with higher *GDF10* expression. This finding could be correlated with the results of a recent study in which GDF10 inhibited PPARγ activity in hepatocytes (30). However, the exact mechanism and clinical implication of the suppression of glucose and lipid homeostasis related to increased expression of GDF10 in SAT could not be explained. Further research is needed to elucidate the regulation of GDF10 secretion and/or the causal or consequent relationship between GDF10 and obesity in humans.

We acknowledge that our study has various limitations. First, the sample size used for RNA sequencing was relatively small. Nevertheless, we obtained six paired samples from each participant who had relatively similar metabolic phenotypes (age 42–59 years, BMI between 20.3–26.9 kg/m^2^) to minimize the inter-individual difference. We also identified DEGs by performing statistical analysis with adjusted *P*-values. However, all six subjects were Asian males, and more studies are needed to extend these findings to women and patients from Western countries. Second, it is not obviously clear whether the serum GDF10 was secreted from SAT. However, a previous study showed that GDF10 secreted by adipocytes is a unique non-covalent complex (28).

In conclusion, we found that *GDF10* expression was higher in SAT than in VAT using RNA sequencing. Bioinformatic analysis showed that the high expression of *GDF10* in SAT was associated with suppression of glucose and lipid metabolism. Furthermore, serum GDF10 concentration was higher in participants with obesity. Therefore, GDF10 may be a SAT-derived adipokine related to obesity. Further studies targeting GDF10 would provide new insight into the development of anti-obesity drugs.

## Data availability statement

The original contributions presented in the study are publicly available. This data can be found here: https://www.ncbi.nlm.nih.gov/geo/query/acc.cgi?acc=GSE231656.

## Ethics statement

The studies involving human participants were reviewed and approved by The Institutional Review Board of Chungnam National University Hospital. The patients/participants provided their written informed consent to participate in this study.

## Author contributions

JL, HK and BK contributed to conception and design. SH, SK, HK, BK and JL provided study materials or patients. MS, JEK, JTK, YK, BK and JL collected and analyzed data. MS and JL wrote the initial draft and revised the manuscript. BK critically reviewed the manuscript. All authors contributed to the manuscript revision, and read and approved the submitted version.
