# Supplementary material for: GDF10 is related to obesity as an adipokine derived from subcutaneous adipose tissue
Source: Front Endocrinol (Lausanne). 2023 Jul 14;14:1159515. doi: 10.3389/fendo.2023.1159515 (PMC10390302; doi:10.3389/fendo.2023.1159515)
Supplement: Supplementary file 1 [file Table_1.docx]

Supplementary Material

GDF10 is related to obesity as an adipokine derived from subcutaneous adipose tissue

Mi Kyung Song^1^, Ji Eun Kim^2^, Jung Tae Kim^2^, Yea Eun Kang^1,2^, Sun Jong Han^3^, Seok Hwan Kim^3^, Hyun Jin Kim^1,2^, Bon Jeong Ku^1,2*^, and Ju Hee Lee^1,2*^

*** Correspondence:** Bon Jeong Ku; [bonjeong@cnu.ac.kr](mailto:bonjeong@cnu.ac.kr) and Ju Hee Lee; [serenaj@cnu.ac.kr](mailto:serenaj@cnu.ac.kr)

**Supplementary Figures and Tables**

Supplementary Table 1. Baseline characteristics of the study participants who underwent adipose tissue biopsy

| Age | Sex | Body mass index (kg/m^2^) | Fasting  Blood  Glucose (mg/dL) | HOMA-IR | Surgical indication |
| --- | --- | --- | --- | --- | --- |
| 42 | Male | 20.3 | 79 | 0.45 | chronic cholecystitis |
| 59 | Male | 22.5 | 93 | 1.40 | Chronic cholecystitis |
| 59 | Male | 26.3 | 97 | 1.72 | Chronic cholecystitis |
| 55 | Male | 24.9 | 65 | 2.18 | Chronic cholecystitis |
| 47 | Male | 24.1 | 105 | 1.97 | Chronic cholecystitis |
| 54 | Male | 24.3 | 94 | 2.32 | Chronic xanthogranulomatous cholecystitis |

HOMA-IR, homeostasis model assessment-insulin resistance
